# Label-free quantitative proteomic analysis of adult *Drosophila* heads

**DOI:** 10.1016/j.xpro.2022.101830

**Published:** 2022-11-11

**Authors:** Yan Zhang, Ioannis P. Nezis

**Affiliations:** 1 State Key Laboratory of Silkworm Genome Biology, Biological Science Research Center, Southwest University, Chongqing 400715, China; 2 School of Life Sciences, University of Warwick, Coventry CV4 7AL, UK

**Keywords:** Cell Biology, Model Organisms, Molecular Biology, Protein Biochemistry, Proteomics, Mass Spectrometry

## Abstract

LIR motif-containing proteins (LIRCPs) bind to LDS (LIR motif docking site) of Atg8-family proteins. In this protocol, we describe steps to identify *Drosophila* LIRCPs, in Atg8a LDS mutants we have created, via label-free quantitative proteomic analysis. We detail steps for extraction of proteins from adult *Drosophila* heads, followed by liquid chromatography–mass spectrometry (LC-MS/MS) analysis. We also describe screening steps of upregulated proteins in Atg8a LDS mutants, leading to identification of novel LIRCPs in *Drosophila*.

For complete details on the use and execution of this protocol, please refer to Rahman et al. (2022).

## Highlights

• A protocol for identifying proteins from adult *Drosophila* heads
• Filter-aided sample preparation procedures are elaborated
• Detailed procedure for MS-based quantitative proteomics is presented
• A detailed presentation of MaxQuant workflow for label-free analysis

## Abstract (editor highlights)

Publisher’s note: Undertaking any experimental protocol requires adherence to local institutional guidelines for laboratory safety and ethics.

## Graphical abstract

**Figure:**
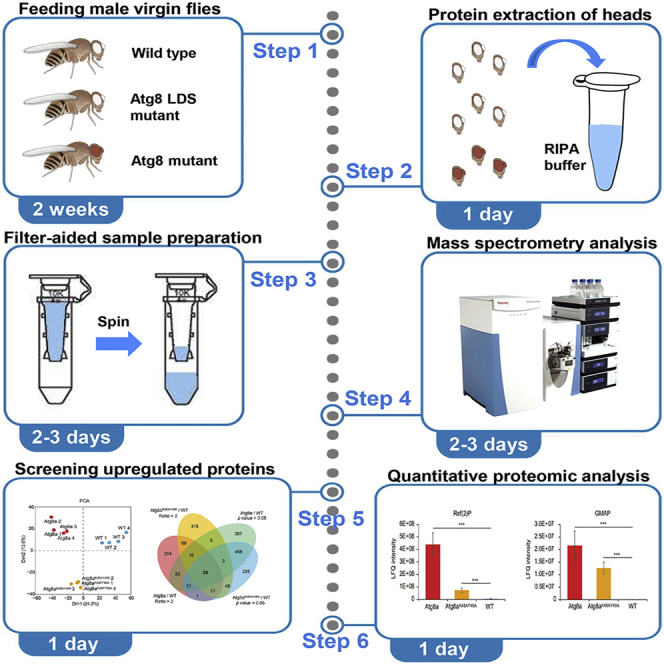

## Before you begin

### Sample collection for quantitative proteomic profiling

**Timing: 4 h**

**Figure 1:**
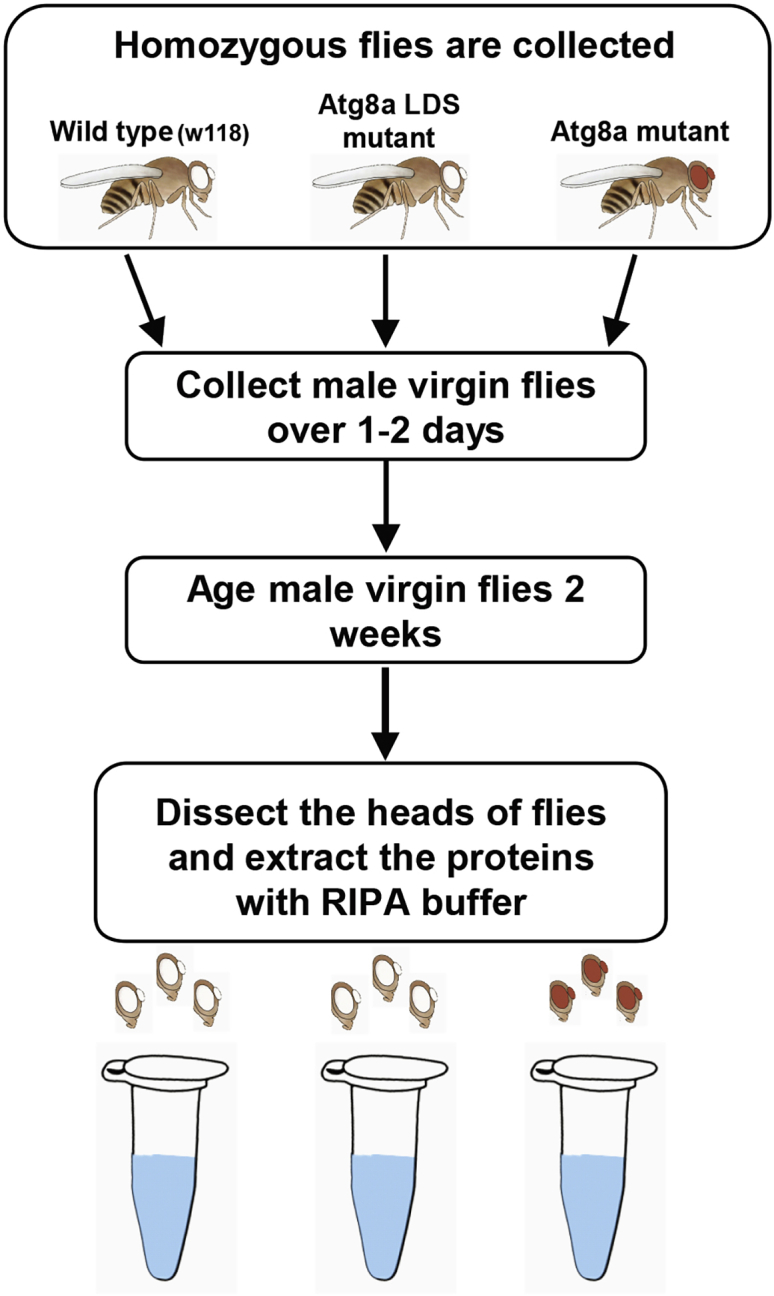
Workflow for collection of adult *Drosophila* heads from wild type and Atg8a mutant flies Atg8a mutant is Atg8a^KG07569^ strain. Atg8a LDS mutant is LIR motif docking site mutant.

This protocol is a comparative proteomic analysis between Atg8a^KG07569^ (Scott et al., 2007), Atg8a LDS (LIR motif docking site) mutants (Rahman et al., 2022) and wild type flies. For experimental uses, homozygous flies are selected and used. Wild type flies ^(w1118)^ are used as controls. Flies are kept at 25°C at 70% humidity in plastic tubes and transferred to new food every 2–3 days. Collect 1–2 days old flies over a 24–36 h period and age for two weeks. Collect two week-old files and use their heads for proteomics analysis. Figure 1 shows the workflow for sample collection.

**CRITICAL:** Only collect male virgin flies. We performed many pre-experiments. We found that the repeatability is very low when we use both male and female fly heads. To improve the repeatability, we only collect male virgin flies.

1. Prepare RIPA buffers and autoclaved pestles in advance.
2. Anaesthetize the flies using 100% CO_2_ by using a CO_2_ blowgun to transmit CO_2_ inside the fly tubes.
3. Transfer flies on a silica pad in a drop of PBS, and isolate heads by using tweezers and micro scissors (neck snap, decapitation).
4. Once you isolate three heads, put them into pre-cooled RIPA buffer (in 1.5 mL Eppendorf tube) by using tweezers. This prevents degradation of the head protein at 25°C.
5. In total, collect 30 heads per sample in 200 μL RIPA buffer.
6. Homogenize samples with a motorized pestle until no debris can be detected, usually 1–2 min. All extraction steps are on ice. To effectively extract proteins, keep samples on ice for 20 min.
7. Centrifuge at 13, 000 g for 20 min at 4°C.

***Note:*** Pre-cool the centrifuge using low speed. Make sure the centrifuge is balanced.

**CRITICAL:** All extraction steps are on ice.

8. Transfer supernatants into new microcentrifuge tubes.
9. Measure protein concentration using Bradford assay method.
  a. Prepare BSA protein standard (1 μg/μL) ahead of time.
  b. Prepare 7 Eppendorf tubes (1.5 mL) for standard curve. Add dye reagent to the tubes according to the table below. Add 799 μL dye reagent to other sample tubes. Protein concentration is measured in duplicate or triplicate.
  c. Add BSA to the tubes according to the table below. Add 1 μL sample to other sample tubes.
    Incubate at 25°C for at least 5 min.
  d. Measure absorbance at 595 nm.
  e. Draw a standard curve. According to the standard curve, calculate the concentration of sample protein.

**Table:** 

BSA standard curve		
BSA (μg)	Dye reagent (μL)	Total (μL)
0	800	800
1	799	800
2.5	797.5	800
5	795	800
10	790	800
15	785	800
20	780	800
10. Store samples at −80°C.

**Pause point:** Samples can be stored at this point for up to 1 month at −80°C.

## Key resources table

**Table:** 

REAGENT or RESOURCE	SOURCE	IDENTIFIER
**Chemicals, peptides, and recombinant proteins**		

Protease Inhibitor Cocktail	Roche	05892791001
Trypsin (MS grade)	Pierce	90058
Acetonitrile (CH_3_CN)	Thermo Scientific	51101
Bradford Reagent	Bio-Rad	5000006
Bovine Serum Albumin	Sigma	A7906
Tris	Sigma	77-86-1
NaCl	Sigma	S3014
Sodium deoxycholate	Sigma	D6750
SDS	Sigma	74255
Igepal	Sigma	I8896
Formic acid (FA; MS grade)	Fluka	56302
M.S. grade water	Fluka	39253
DTT	Sigma	D9163
IAA	Sigma	I1149
Ammonium Bicarbonate (NH_4_HCO_3_)	Fluka	40867
Urea	Sigma	51456

**Experimental models: Organisms/strains**		

Wild Type (WT)	WellGenetics	*w[1118]*
Atg8a ^KG07569^	Bloomington #14639	*Atg8a [KG07569]/FM7c*
Atg8a LDS mutant	WellGenetics	*w[1118], Atg8a K48A Y49A CRISPR/FM7a*

**Software and algorithms**		

MaxQuant	Tyanova et al. (2016)	MaxQuant v1.6.5.0

**Other**		

Tweezers	Dumont	Dumostar #55
Drosophila Tubes 25 × 95 mm	Flystuff	F18298
CO_2_ Blowgun	Flystuff	FLY1042
Micro Scissor	Surtex® Vannas	Angled 0.1 mm tip
0.5 Centrifugal filter unit	Millipore	UFC500396
Table Top Centrifuge	Eppendorf	5417R
Speedvac concentrator	Labconco	Centrivap
RSLC nano HPLC system	Thermo Scientific	UliMate^TM^ 3000
Nano-LC precolumn	Thermo Scientific	160454
Nano-LC analytical column	Thermo Scientific	164540
Mass Spectrometry	Thermo Scientific	Orbitrap Fusion

## Materials and equipment

**Table:** Lysis buffer (RIPA)

Reagent	Final concentration	Amount
Tris HCl (pH 7.4) (1 M stock)	50 mM	5 mL
NaCl (5 M stock)	150 mM	3 mL
Igepal	1%	1 mL
Sodium deoxycholate	0.5%	0.5 g
SDS (10% stock)	0.1%	1 mL
ddH_2_O	N/A	90 mL
**Total**	**N/A**	**100 mL**

**CRITICAL:** RIPA buffer can be stored at 4°C, for a maximum of 6 months. Before use, add one tablet of protease inhibitor cocktail per 10 mL RIPA buffer. Once adding protease inhibitor, it can be stored at 4°C, for less than 1 week.

***Note:*** Igepal, sodium deoxycholate are harmful if swallowed. Igepal causes skin irritation and serious eye damage. SDS is flammable solid and harmful if swallowed or if inhaled. When you are preparing the RIPA buffer, wear eye and face protection and keep away from open flame.

**Table:** Urea (UA) buffer

Reagent	Final concentration	Amount
Tris	50 mM	181.5 mg
NaCl	75 mM	131.4 mg
Urea	8 mM	14.4 g
ddH_2_O	N/A	up to 30 mL
**Total**	**N/A**	**30 mL**

***Note:*** Initially, add all the reagents to 20 mL of water. Make the volume to 30 mL when all reagents are dissolved.

***Note:*** UA buffer can be stored at 25°C for 3 days (Prepare fresh before beginning the experiment).

**Table:** ABC buffer

Reagent	Final concentration	Amount
Ammonium bicarbonate	50 mM	197.6 mg
ddH_2_O	N/A	up to 50 mL
**Total**	**N/A**	**50 mL**

***Note:*** ABC buffer can be stored at 25°C for 1 week.

**Table:** Reduction buffer (DTT buffer)

Reagent	Final concentration	Amount
DTT	1 M	0.0154 g
UA buffer	N/A	1 mL
**Total**	**N/A**	**1 mL**

**Table:** Alkylation buffer (IAA buffer)

Reagent	Final concentration	Amount
IAA	1 M	0.0185 g
UA buffer	N/A	1 mL
**Total**	**N/A**	**1 mL**

***Note:*** DTT and IAA stocks are stored at −20°C, for a maximum of 6 months. IAA should be weighed in the fume hood.

***Note:*** DTT and IAA are harmful if swallowed. DTT causes skin irritation and serious eye damage. IAA may cause an allergic skin reaction. Wear eye and face protection and prevent release to the environment when you are preparing.

**Table:** Trypsin buffer

Reagent	Final concentration	Amount
Trypsin	20 μg/mL	20 μg
ABC buffer	N/A	1 mL
**Total**	**N/A**	**1 mL**

***Note:*** Trypsin buffer can be aliquoted and stored at −80°C, for a maximum of 6 months.

**Table:** LC Solvent A

Reagent	Final concentration	Amount
Formic acid	0.1%	25 μL
Milli Q water	N/A	25 mL
**Total**	**N/A**	**25 mL**

**Table:** LC Solvent B

Reagent	Final concentration	Amount
Formic acid	0.1%	25 μL
Acetonitrile	99.9%	25 mL
**Total**	**N/A**	**25 mL**

**CRITICAL:** Degassing of LC solvent for 30 min using ultrasound, and then add formic acid. This prevents formic acid volatilization.

***Note:*** LC solvent can be stored at 25°C for 1 week.

***Note:*** Formic acid and acetonitrile are flammable liquid. Both them are harmful if swallowed. Keep away from heat, hot surfaces, sparks, open flames and other ignition sources. No smoking.

## Step-by-step method details

### Quantitative proteomics analysis of adult *Drosophila* heads

**Timing: 2 days**

In this study, we use two autophagy mutants: Atg8a^KG07569^ (Scott et al., 2007) and Atg8a LDS (LIR motif docking site) mutant (Rahman et al., 2022). It is hypothesized that these flies have defective LIR motif binding ability and thus accumulate LIR motif containing proteins. We perform label-free quantitative proteomic to analyze the accumulated proteins in Atg8a LDS, Atg8a mutant compare to wild type flies. The accumulated proteins are putative selective autophagy receptors.

**Figure 2:**
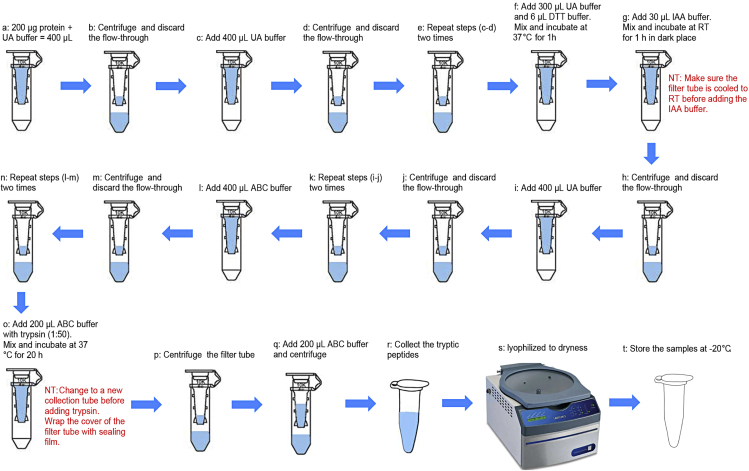
Workflow for tryptic digestion of adult *Drosophila* head proteins a-b: Load samples to the filter tubes; c-e: Wash samples with UA buffer to remove RIPA buffer; f-g: Reduction and alkylation with DTT and IAA buffer; h-k: Wash samples with UA buffer to remove DTT and IAA buffer; l-n: Wash samples with ABC buffer to remove UA buffer; o: Digest samples with trypsin buffer; p-r: Collect the tryptic peptides from the filter tubes; s-t: Evaporate the samples to dryness.

1. Sample preparation for Label-Free-Quantification (LFQ) based mass spectrometry analysis.
  a. In this protocol, we use 200 μg of protein extraction for digestion.
    i. Calculate the required volume of 200 μg protein according to the protein concentration.
    ii. Mix 200 μg of protein extraction with UA buffer in the filter tube (Millipore, UFC500396).
    iii. Make sure the total volume is 400 μL.
  b. Centrifuge the filter units at 12, 000 g for 20 min at 4°C and discard the flow-through from the collection tube.
  c. Add 400 μL of UA buffer to the filter tube.
  d. Centrifuge at 12, 000 g for 20 min at 4°C. Discard the flow-through from the collection tube.
  e. Repeat steps (c-d) two times.
  f. Add 300 μL of UA buffer and 6 μL of 1 M DTT buffer to the filter tube. Mix and incubate at 37°C for 1 h.
  g. Add 30 μL of 1 M IAA buffer to the filter tube. Mix and incubate at 25°C for 1 h.
    **CRITICAL:** Make sure the filter tube is cooled to 25°C before adding the IAA buffer.
    ***Note:*** Incubate in dark place.
    ***Alternatives:*** Reduction and alkylation can also be use 10 mM TCEP (Tris(2-carboxyethyl)phosphine) and 40 mM CAA (Cyanoacetamide) in ABC buffer for 30 min at 25°C.
  h. Centrifuge at 12, 000 g for 20 min at 4°C. Discard the flow-through from the collection tube.
  i. Add 400 μL of UA buffer to the filter tube.
  j. Centrifuge at 12, 000 g for 20 min at 4°C. Discard the flow-through from the collection tube.
  k. Repeat steps (i-j) two times.
  l. Add 400 μL of ABC buffer to the filter tube.
  m. Centrifuge at 12, 000 g for 20 min at 4°C. Discard the flow-through from the collection tube.
  n. Repeat steps (l-m) two times.
  o. Add 200 μL ABC buffer with trypsin (enzyme to protein ratio 1: 50), incubate at 37°C for 20 h.
    ***Note:*** Change to a new collection tube before adding trypsin.
    ***Note:*** When incubating, wrap the lid of the filter tube with sealing film.
  p. Centrifuge the filter units at 12, 000 g for 20 min at 4°C.
  q. Add 200 μL ABC buffer and centrifuge the filter units at 12, 000 g for 20 min at 4°C.
  r. Collect the tryptic peptides from the collection tube.
  s. Evaporate the samples to dryness using a Labconco Speedvac (Centrivap, Labconco, USA), usually for 3–4 h.
  t. Store the samples at −20°C until use.
    A workflow of sample preparation for Mass spectrometry is shown in Figure 2.
    **CRITICAL:** All biological repeats are digested at the same time to improve the repeatability. If your repeats are digested in batches, make sure you use the same materials and methods.
    **Pause point:** Samples can be stored at this point for up to 1 month at −80°C.

### Mass spectrometry and statistical analysis

**Timing: weeks**

Tryptic peptides are analyzed by mass spectrometry to identify the accumulated proteins in Atg8a^KG07569^, Atg8a LDS mutant compared to wild type flies.

2. Samples are analyzed by mass spectrometry.
  a. Prepare LC-MS/MS equipment.
    i. Both MS and MS/MS calibration should be carried out at regular intervals (monthly and at the beginning of a new batch of an experiment).
    ii. Install fresh mobile solvent A and solvent B in sufficient amounts.
    iii. Install the precolumn and analytical chromatography column.
    iv. Flush air of pump A, pump B and pump S with mobile phase buffer. The flush threshold is 10 μL.
    v. Equilibrate the precolumn and analytical column with solvent A buffers.
    vi. Check LC-MS/MS performance by running a blank sample. Ensure the mobile phase flow path is smooth.
    vii. It is highly recommended to use quality control samples such as mixtures of standards to check if the LC-MS/MS performs as expected.
  b. Prepare the samples.
    i. Dissolve tryptic peptides in 200 μL of 0.1% formic acid (solvent A).
    ii. Centrifuge peptides at 12, 000 g for 20 min at 4°C.
    iii. Transfer the supernatant to a new Eppendorf tube.
  c. Load 10 μL samples into an Ultimate 3000 RSLCnano HPLC (Dionex) using an Acclaim PepMap μ-precolumn catridge (C18, 5 μm, 100 Å, 300 μm × 5 mm) and an analytical Acclaim PepMap RSLC column (C18, 2 μm, 100 Å, 75 μm × 50 cm, Thermo Scientific).
  d. LC gradient and LC parameter settings are in the table below.

**Table:** 

LC gradient profile			
Time	Duration	Flow (nL/min)	%B
0	0	250	3
8	8	250	3
140	132	250	25
163	23	250	90
165	2	250	3
180	15	250	3
    **Table:** 

LC parameter settings	
Injection volumn	10 μL
Column temperature	40°C
Sampler temperature	6°C
Max pressure limit	480 Bar
  e. Peptides are subjected to Nano Spray ionization (NSI) source followed by tandem mass spectrometry (MS/MS) in Thermo Orbitrap Fusion coupled online to UPLC (Ultimate 3000 RSLCnano HPLC) (Dionex) or Q exactive coupled online to EASY-nLC 1000 system.
  f. The electrospray voltage applied is 2.1 kV.
  g. The m/z scan range is 375–1,575 m/z for full scan, and intact peptides are detected in the Orbitrap at a resolution of 120,000 resolution with a 2 × 10^5^ ion count target.
  h. The maximum injection time is set to 150 ms.
  i. Tandem MS is performed with an isolation window at 1.2 Th using the Quadrupole. Use of wider isolation windows improves sensitivity, but noise of ions also increases. We set isolation window at 1.2 Th for Orbitrap Fusion Mass Spectrometer, 2.2 m/z for Q exactive Mass Spectrometer according to our experience.
  j. HCD (High-energy collisional dissociation) is significantly affected by the normalized energy applied. HCD fragmentation with normalized collision energy of 33 or 32 is recommended for Orbitrap Fusion Mass Spectrometer, 27 for Q exactive Mass Spectrometer.
  k. The MS^2^ ion count target is set to 5 × 10^3^ and maximum injection time is 200 ms. Precursors with charge state 2–7 are selected and sampled for MS^2^.
  l. The dynamic exclusion duration is set to 50 s with a 10 ppm tolerance around the selected precursor and its isotopes. Fixed first mass is set as 120 m/z.
  m. Monoisotopic precursor selection is turned on and instrument is run in top speed mode.

**Table:** 

Orbitrap fusion method summary	
**Global settings**	

Method Duration (min)	180
Ion Source Type	NSI
Spray Voltage: Positive Ion (V)	2100
Ion Transfer Tube Temp (°C)	275
Default Charge State	1

**Full Scan - MS1**	

Detection	Orbitrap
Resolution	120 K
Scan Range (m/z)	375–1,575
Max Injection Time (ms)	150
AGC Target	200 000
DataType	Profile

**Filter Settings**	

Include Charge State (s)	2–7
Exclusion Duration (s)	50
Mass tolerance (ppm)	10

**dd scan – MS2**	

Isolation Mode	Quadrupole
Isolation Window	1.2
FirstMass	120
ActivationType	HCD
Collision Energy (%)	33
Detector Type	Iontraps
Ion trap scan rate	Rapid
Max Injection Time (ms)	200
AGC Target	5 000
3. MaxQuant analysis of Samples.
  All acquired raw data are searched against *Drosophila melanogaster* database and the common contaminant database by MaxQuant software (v1.6.5.0) (Cox et al., 2011; Tyanova et al., 2016). Please see Figure 3 for workflow on MaxQuant detailed parameters for analysis of these data. Unless explicitly stated, parameters in MaxQuant have not been changed from their standard values.
  a. Along the top of MaxQuant are six tabs. Select **Raw data**, then click “Load” to load all raw data (step 1).
  b. Write template (step 2), then a txt named “experimentalDesignTemplate” will automatically appear in the folder. Assign the experiment number (step 3) and then read the “experimentalDesignTemplate” from file (step 4).
  c. In the **Group-specific parameters tab**, choose type as ”Standard” and the “Multiplicity” is 1 for label-free quantification (step 5). Choose “Multiplicity” 2 if you have light and heavy labels, and 3 if you have light, medium, and heavy.
  d. Precursor mass tolerance is 4.5 ppm and product ions are searched at 15 ppm tolerances (steps 6 and 10).
  e. Peptides are generated from a tryptic digestion with up to two missed cleavages (step 7), carbamidomethylation of cysteines as fixed modifications, and oxidation of methionine as variable modifications.
  f. We use LFQ with minimum ratio count of 2 to perform label-free quantification (step 8).
  g. On the **Global parameters** tab, click “sequence” and add a FASTA file of *Drosophila* proteome downloaded from Uniport (step 9).
  h. Minimum peptide length is set at 7, while the estimated false discovery rate (FDR) threshold for peptide and protein are specified at maximum 1% (step 11).
    **CRITICAL:** Perform three or four replicates for Atg8a, Atg8a-LDS mutant and wild type in order to improve the accuracy of results.

**Figure 3:**
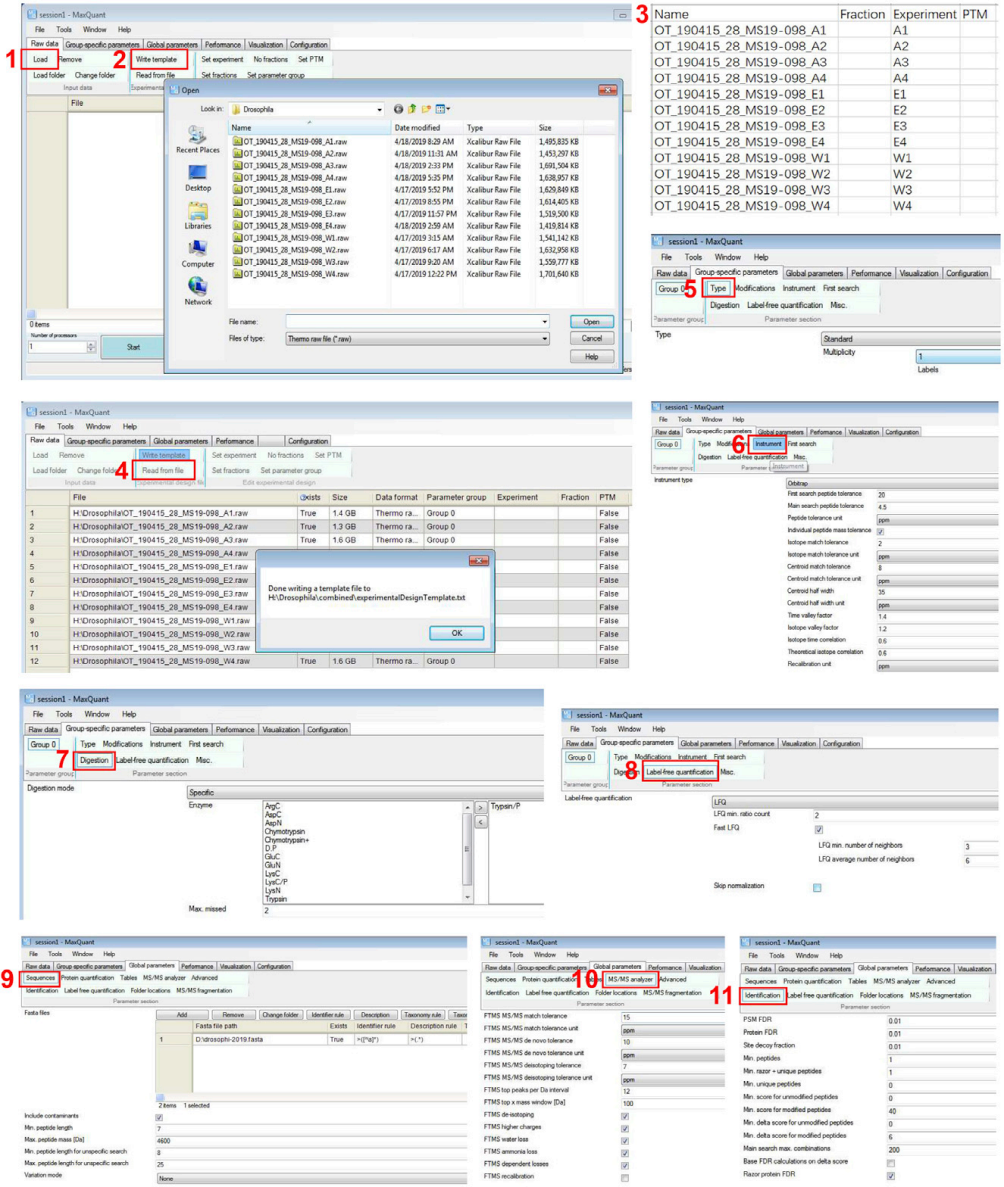
Screenshots of MaxQuant workflow for identifying proteins
4. Identify the accumulate proteins.
  a. All result files appear in the folder “…\combined\txt” as tab-delimited text files. Data processing and annotation are performed by manual operation.
  b. Open “ProteinGroups” with Microsoft Excel, and remove the reverse and contaminant hits (as defined in MaxQuant) from the MaxQuant output files.
  c. Only protein groups identified with at least one unique peptide are used for the analysis.
  d. Calculate the average LFQ intensity of each protein group in each type of sample.
  e. The ratio is calculated by dividing the average value of LFQ intensity of Atg8a, Atg8a LDS mutant sample by average value of LFQ intensity of wild type sample (Atg8a/ wild type, Atg8a LDS/ wild type).
  f. Protein groups are assigned a probability value (p-value) using a two-sample Student’s T-Test.
  g. Proteins are considered significant if the p-value *<* 0.05 and had a more than two-fold change in protein expression.

## Expected outcomes

**Figure 4:**
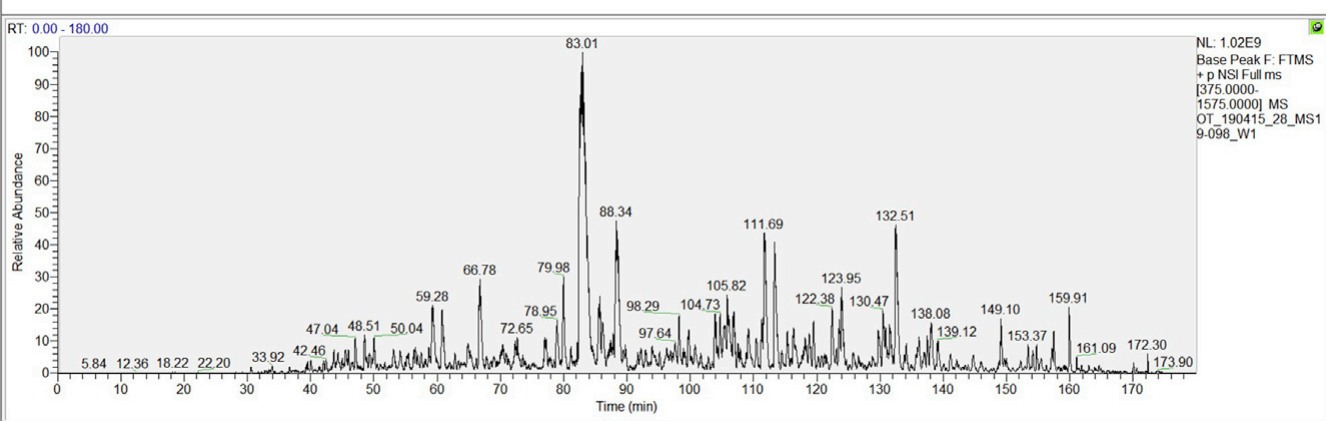
Representative chromatogram of a sample showing the relative abundance at the y axis and retention time at the x axis

**Figure 5:**
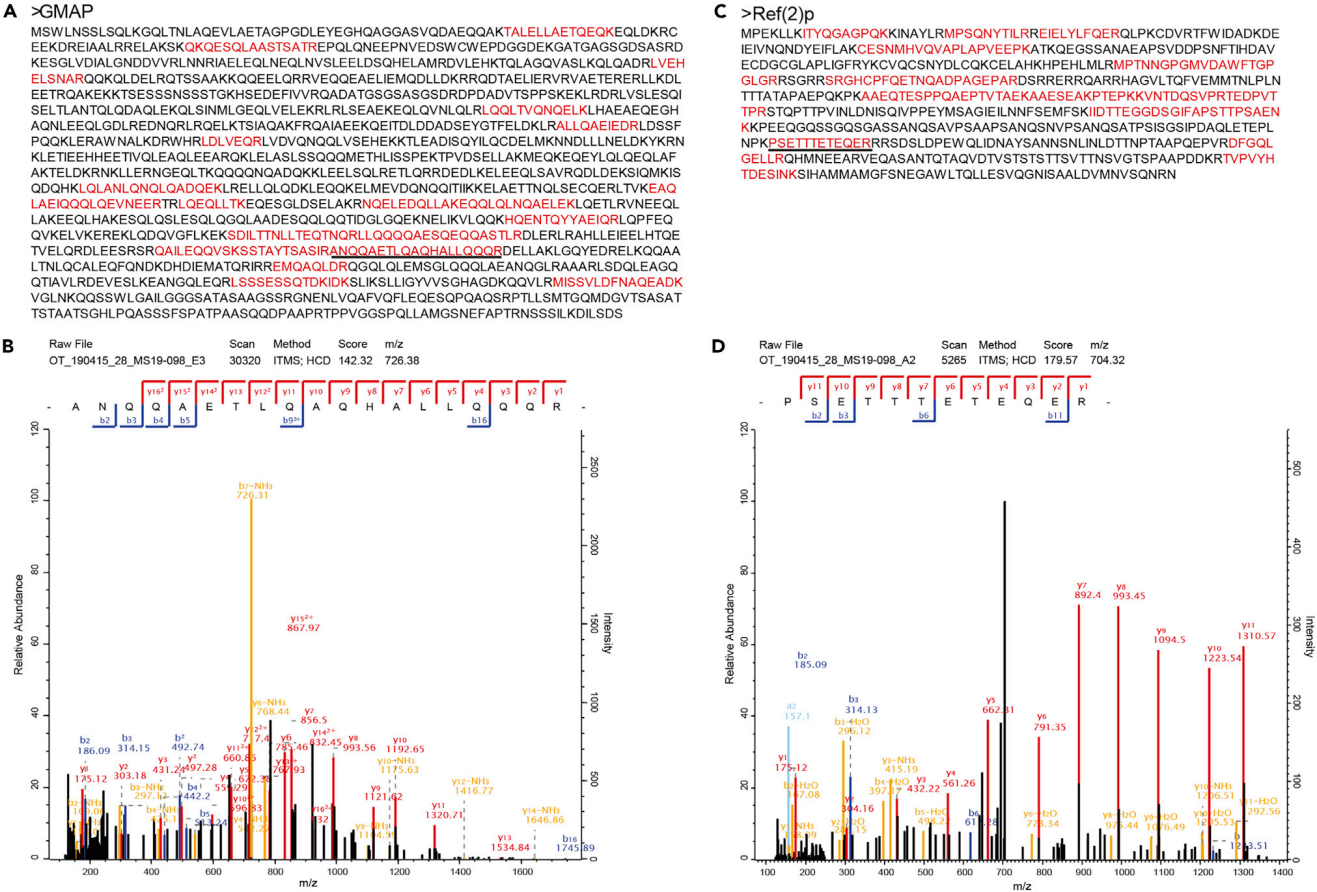
Identified peptides of Ref(2)p and GMAP and their annotated tandem mass spectra (A) All identified peptides for GMAP are marked in red color. The annotated tandem mass spectra of the underlined sequence is shown in (B). (B) The annotated tandem mass spectra of one peptide for GMAP is extracted by using MaxQuant viewer software. (C) All identified peptides for Ref(2)p are marked in red color. The annotated tandem mass spectra of the underlined sequence is shown in (D). (D) The annotated tandem mass spectra of one peptide for Ref(2)p is extracted by using MaxQuant viewer software.

Mass spectrometry-based comparative proteomic study helps efficiently to identify the accumulated proteins in Atg8a and Atg8a LDS mutant fly heads compared to wild type. Thus, a successful MS-based assay can provide high quality data. Figure 4 shows a representative chromatogram of wild type fly heads. X axis represents the retention time, and y axis represents the relative abundance. In this study, we identified 3036, 2342, 2468 proteins from wild type, Atg8a ^KG07569^ and Atg8a-LDS mutants, respectively. All the accumulated proteins with a difference of more than 2-fold between mutant and wild type flies are included in the below table. Figure 5 shows the all identified peptides of Ref(2)p and GMAP. We can see many peptides are identified, indicating the confidence is high. The annotated tandem mass spectra of two peptides are extracted by using MaxQuant viewer software, as shown in Figure 5.

**Table:** Accumulated proteins in autophagy mutant flies

Proteins	Peptides	Unique peptides	Ratio AM/W	Ratio LM/W	p value AM/W	p value LM/W	Preferred name	LIR motif PSSM>13	Anchor
tr|Q7K3E2	22	22	2.68	2.02	0.011592959	0.035571810	CG5080	YES	NO
tr|A0A0B4K6W2	18	18	∞	∞	0.000007578	0.031221334	faf	YES	NO
tr|Q6IHY5	2	2	∞	∞	0.000200425	0.028880452	CG34172	NO	NO
tr|Q8IR72	3	3	∞	∞	0.030183763	0.024545484	CG32638	NO	NO
tr|Q9VDU7	7	7	4.47	4.03	0.014600354	0.024337065	Naam	NO	NO
tr|Q9VLV9	2	2	3.88	2.43	0.001153117	0.023807031	Proc	NO	NO
tr|A0A0B4KEK7	4	4	2.03	2.09	0.049797907	0.021273536	PI31	YES	NO
tr|Q9VIX4	20	20	8.85	4.05	0.002104985	0.018795974	CG17544	YES	NO
tr|Q9VZF1	9	9	3.77	3.53	0.007762399	0.012796017	CG1309	NO	NO
sp|P02515	9	9	28.79	4.29	0.001372423	0.007079160	Hsp22	YES	NO
tr|A0A0B4LH23	8	8	4.55	5.72	0.013546870	0.005457873	RIC-3	NO	NO
tr|Q9VGE7	9	9	2.40	3.11	0.013959163	0.004790721	Ect3	NO	NO
tr|Q7K3B7	14	14	5.89	4.39	0.003005442	0.002326007	CG11208	YES	NO
tr|Q7K5M6	9	9	8.10	7.44	0.001478584	0.001739584	Sip1	NO	YES
sp|Q9VG97	7	7	15.47	13.47	0.000945625	0.000784903	GstD3	YES	NO
tr|Q53XG2	7	7	∞	∞	0.000004069	0.000449452	dally	YES	YES
tr|Q9VBU6	6	1	2.41	2.03	0.000069108	0.000258626	CG11857	YES	NO
sp|P14199	17	17	99.73	16.78	0.000094014	0.000198945	Ref(2)P	YES	YES
sp|Q9VIU7	5	5	∞	∞	0.000000013	0.000170861	CG10166	YES	NO
tr|Q9W3Q1	9	9	∞	∞	0.037598688	0.000045288	Pdp	YES	NO
tr|Q9VXU2	21	21	∞	∞	0.000306313	0.000042015	GMAP	YES	YES
tr|M9PCN6	6	6	∞	∞	0.000097701	0.000020478	numb	NO	NO
tr|Q9VG92	6	6	∞	∞	0.000004919	0.000017222	GstD8	YES	NO
tr|Q9VN39	5	5	∞	∞	0.031306116	0.000009126	CG9775	NO	YES
tr|A1ZB69	9	9	2.20	3.12	0.000851777	0.000008333	GstE4	YES	NO
tr|A4V488	7	7	∞	∞	0.032566158	0.000006712	ras	YES	YES
tr|A0A0B4JD21	8	8	∞	∞	0.000000154	0.000005614	CG10253	NO	NO
tr|Q86BQ3	8	7	∞	∞	0.000019801	0.000003868	CG13284	YES	NO
tr|A0A0B4LGN1	18	18	∞	∞	0.000000004	0.000000066	Itp-r83A	YES	NO

## Limitations

This protocol enables identification of accumulated proteins in Atg8a ^KG07569^ and Atg8a-LDS mutant *Drosophila* heads compared to wild type. In this protocol, we use the fly head because the brain is relatively small and difficult to dissect out to extract enough proteins for proteomics.

## Troubleshooting

### Problem 1

Our project is to identify accumulated proteins in autophagy mutants. In our pre-experiments, we used methanol precipitation technique to extract proteins and in-solution digestion method. Although Ref(2)p is a well-studied LIRCP, it was not identified in the pre-experiments (step for sample collection for quantitative proteomic profiling).

### Potential solution

We thought methanol precipitation and in-solution digestion techniques may not have extracted some proteins. Then, we used RIPA buffer to extract proteins and Filter Aided Sample Preparation (FASP) method to digest proteins. More proteins were identified.

### Problem 2

For the second pre-experiments, we collected whole flies as our samples. The number of identified proteins was very low (step for sample collection for quantitative proteomic profiling).

### Potential solution

We speculated that protein composition is very diverse in whole flies. The intensity of high-abundant proteins may inhibit the low-abundant proteins. We collected fly heads to perform quantitative proteomic analysis. In addition, we increased nano-LC gradient time from 120 min to 180 min. We increased the protein number from 1843 to 2528 proteins.

### Problem 3

User may find that the chromatogram peaks are discontinuous and have low relative intensity during MS analysis (step 3).

### Potential solution

Check the spray needle (nano-bore emitters), and use a new one if necessary. Increase of sample loading can increase the intensity.

### Problem 4

In the pre-experiments, the repeatability of proteomics was poor (step 4).

### Potential solution

In the pre-experiments, we collected both male and female fly heads. To improve the repeatability, we only used the male virgin flies in the experiments. In addition, all repeat samples are digested at the same time. Perform the mass spectrometry for all repeat samples within one or two days.

### Problem 5

There are many accumulated proteins identified in Atg8a ^KG07569^ and Atg8a LDS mutant compared to wild type (step 4).

### Potential solution

Select the accumulated proteins in both Atg8a ^KG07569^ and Atg8a LDS mutants by using the cut-off p-value as less than 0.05 together with a difference of more than two-fold between mutant and wild-type flies. Choose the proteins which have putative LIR motifs by using the iLIR software (Jacomin et al., 2016).

## Resource availability

### Lead contact

Further information and requests for resources and reagents should be directed to and will be fulfilled by the lead contact, Ioannis P. Nezis (I.Nezis@warwick.ac.uk).

### Materials availability

This study did not generate any new unique reagents and/or materials.

## Data Availability

This study did not generate new unique datasets or codes.
